# Correction: Steroid hormones induce *bcl-X* gene expression through direct activation of distal promoter P4

**DOI:** 10.1016/j.jbc.2021.100689

**Published:** 2021-04-28

**Authors:** Luciana Rocha-Viegas, Guillermo P. Vicent, José L. Barañao, Miguel Beato, Adalí Pecci

VOLUME 279 (2004) PAGES 9831–9839

The first author of the above article is incorrectly identified as “Luciana Rocha Viegas.” The correct name is “Luciana Rocha-Viegas.”

